# Effects of Brain Pathologies on Spatiotemporal Gait Parameters in Patients with Mild Cognitive Impairment

**DOI:** 10.3233/JAD-221303

**Published:** 2023-10-24

**Authors:** Magnus Lindh-Rengifo, Stina B. Jonasson, Susann Ullén, Sebastian Palmqvist, Danielle van Westen, Erik Stomrud, Niklas Mattsson-Carlgren, Maria H. Nilsson, Oskar Hansson

**Affiliations:** a Department of Health Sciences, Faculty of Medicine, Lund University, Lund, Sweden.; b Memory Clinic, Skåne University Hospital, Malmö, Sweden; c Clinical Studies Sweden – Forum South, Skåne University Hospital, Lund, Sweden; d Department of Clinical Sciences Malmö, Clinical Memory Research Unit, Lund University, Lund, Sweden; e Diagnostic Radiology, Clinical Sciences Lund, Lund University, Lund, Sweden; f Image and Function, Skåne University Hospital, Lund, Sweden; g Department of Neurology, Skåne University Hospital, Lund, Sweden; h Wallenberg Centre for Molecular Medicine, Lund University, Lund, Sweden

**Keywords:** Alzheimer’s disease, Alzheimer’s disease pathology, amyloid-β, electronic walkway, gait, gait variability, mild cognitive impairment, tau, white matter hyperintensities

## Abstract

**Background:**

Impaired gait can precede dementia. The associations between gait parameters and brain pathologies are therefore of interest.

**Objective:**

To explore how different brain pathologies (i.e., vascular and Alzheimer’s) are associated with specific gait parameters from various gait components in persons with mild cognitive impairment (MCI), who have an increased risk of developing dementia.

**Methods:**

This cross-sectional study included 96 patients with MCI (mean 72, ±7.5 years; 52% women). Gait was evaluated by using an electronic walkway, GAITRite^®^. Four gait parameters (step velocity variability; step length; step time; stance time asymmetry) were used as dependent variables in multivariable linear regression analyses. Independent variables included Alzheimer’s disease pathologies (amyloid-β and tau) by using PET imaging and white matter hyperintensities (WMH) by using MRI. Covariates included age, sex, comorbidities (and intracranial volume in analyses that includedWMH).

**Results:**

Increased tau-PET (Braak I–IV region of interest [ROI]) was associated with step velocity variability (standardized regression coefficient, β= 0.383, *p* < 0.001) and step length (β= 0.336, *p* < 0.001), which remained significant when using different Braak ROIs (I-II, III-IV, V-VI). The associations remained significant when adjusting for WMH (*p* < 0.001). When also controlling for gait speed, tau was no longer significantly (*p* = 0.168) associated with an increased step length. No significant associations between gait and Aβ-PET load or WMH were identified.

**Conclusions:**

The results indicate that one should pay specific attention to assess step velocity variability when targeting single task gait in patients with MCI. Future studies should address additional gait variability measures and dual tasking in larger cohorts.

## INTRODUCTION

Gait can be divided into different components such as pace, rhythm, variability, and asymmetry; each component includes various gait parameters (e.g., gait speed and step length have been identified in a pace focused component) [1–3]. Decreased gait speed has been associated with cognitive impairment in cross-sectional studies [4, 5], which might reflect that both functions share similar brain regions or networks [4]. Interestingly, decreased gait speed precedes cognitive decline [4], and gait impairments precede the two most common major neurocognitive disorders (“dementia”), i.e., Alzheimer’s disease (AD) and vascular dementia [6, 7]. That is, other gait parameters than gait speed can also be of diagnostic value [8–10]. It is therefore of interest to investigate how brain pathologies are associated with specific gait parameters in individuals with an increased risk of developing dementia.

Individuals with mild cognitive impairment (MCI) have an increased risk of progressing to dementia [11], but not everyone with MCI develops dementia. The most common cause of progressing from MCI to dementia, is having an underlying AD pathology and the second most common cause is vascular pathology [12]. Alzheimer’s pathology includes accumulation of amyloid-β plaques (Aβ) and tau tangles [13], which can be measured using positron emission tomography (PET) or cerebrospinal fluid (CSF). Signs of having an incipient AD include having an abnormal CSF Aβ_42/40_ ratio [14], whereas signs of vascular burden include white matter hyperintensities (WMH; measured using magnetic resonance imaging [MRI]); WMH relate to dementia [15, 16].

A review of cross-sectional studies reported that the majority of studies found an association between increased WMH and slow gait speed in older adults, but it highlighted that there is knowledge gaps in relation to several gait parameters (e.g., gait speed variability, swing time asymmetry, and stance time asymmetry) [17]. Sakurai et al. showed that WMH were only associated with stride length variability in individuals with mild or no cognitive impairment. Among those with more severe cognitive impairments, WMH were associated with both gait speed and several variability measures [18].

Increased Aβ burden (assessed with PET imaging) has been associated with gait impairments (decreased gait speed [19–22], increased variability [19, 23], reduced cadence [19], and increased double support time [19]) in older adults without dementia. Total tau-level (CSF) has been associated with the gait component rhythm (i.e., in a dual task context [24]) and gait speed [25] in individuals both with and without cognitive impairment. To our knowledge, no study has investigated the independent effects of tau, Aβ, and WMH on gait parameters from various components of gait in individuals with MCI.

This explorative study aims to investigate how different brain pathologies (i.e., WMH, Aβ, and tau pathology) independently relate to objective gait parameters from various gait components in patients with MCI (with signs of an incipient neurodegenerative disorder, i.e., with an increased risk of developing dementia). More specifically, we investigated the gait parameters step velocity variability, step length, step time, and stance time asymmetry.

## METHODS

This study is part of Motor-ACT (Motor aspects and activities in relation to cognitive decline), which is a sub-study to the larger BioFINDER-2 study (Biomarkers for identifying neurodegenerative disorders early and reliably, NCT03174938).

### Participants

This study included patients with MCI with signs of an incipient neurodegenerative disorder (see below), who were recruited from the Memory Clinic at Skåne University Hospital, Sweden.

Inclusion and exclusion criteria for the BioFINDER-2 study have been described previously, e.g., NCT03174938, [26, 27]. The patients with MCI had the following inclusion criteria: 40–100 years of age; they were referred to the memory clinic due to cognitive symptoms (self-experienced or experienced by informant); based on clinical assessments, CSF analyses and imaging results, the medical doctor interpreted that the cognitive symptoms were caused by an incipient neurodegenerative disorder (e.g., abnormal CSF ratio of Aβ_42/40_ or any core criteria of prodromal dementia with Lewy bodies [28]); Mini-Mental State Examination (MMSE) score was ≥24 at the BioFINDER-2 baseline visit; Speaks and understands Swedish sufficiently, and the patient did not fulfill the criteria for dementia as defined by the Diagnostic and Statistical Manual of Mental Disorders 5th revision (DSM-5) [29]. The participants were categorized as having MCI if scoring worse than –1.5 z-score in at least one cognitive domain (memory, verbal, attention/executive and visuospatial) at the baseline visit as compared to healthy controls (adjusted for age and education when significant) [30]. Memory was assessed using the 10-word delayed recall test from ADAS-cog. Verbal ability was assessed using animal fluency and the 15-item short version of the Boston Naming Test.

Attention and executive function were assessed by using the Trail Making Test A and B (TMT), and the Symbol Digit Modalities Test (SDMT). Visuospatial ability was measured by using incomplete letters and cube analysis from the Visual Objects and Space Perception (VOSP) battery.

General exclusion criteria were refusing PET, MRI or lumbar puncture, ongoing significant misuse of alcohol or substances, or difficulties to participate due to unstable systemic illness.

The current study had additional exclusion criteria: using mobility devices during gait assessments (*n* = 7) or that less than 30 steps were registered by the software for gait assessments (*n* = 6) [31]. Eight persons converted to dementia prior to gait assessments according to their treating physician and were thus excluded (the time delay between inclusion in BioFINDER-2 and gait assessments was in mean 296 [SD 47] days). Finally, 18 persons were excluded due to missing data on either PET or MRI. Consequently, the study sample included 96 patients with MCI. The mean (SD; min-max) age was 72 (7.5; 45–93) years and 52.1% were women. Descriptive data of participant characteristics, independent variables, covariates, and the included gait parameters (i.e., dependent variables) are presented in Table 1.

**Table 1:** Participants’ characteristics and descriptive data

Variable	Mild cognitive impairment, *n* = 96	Missing, n
Age (y), mean (SD)	72.4 (7.5)	–
Sex (woman), *n* (%)	50 (52.1%)	–
Educational level (y), median (q1–q3)	12.0 (9.0–14.0)	1
BMI (kg/m^2^), mean (SD)	25.6 (3.6)	–
Leg length (cm), mean (SD)^a^	87.8 (5.7)	–
Concerns about falling (FES-I), mean (SD)	20.0 (6.1)	1
Amnestic mild cognitive impairment (yes), *n* (%)	66 (70.2%)	2
Attentional/Executive mild cognitive impairment (yes), *n* (%)	43 (44.8%)	–
Verbal mild cognitive impairment (yes), *n* (%)	21 (22.1%)	1
Visuospatial mild cognitive impairment (yes), *n* (%)	9 (9.9%)	5
History of stroke/transient ischemic attack (yes), *n* (%)	6 (6.3%)	–
Diabetes (yes), *n* (%)	13 (13.5%)	–
Intracranial volume (mL), mean (SD)	1503.3 (144.9)	–
Gait velocity (m/s), mean (SD)	1.1 (0.2)	–
*Gait parameters (dependent variables)*
Step velocity variability (cm/s), mean (SD)	6.3 (2.0)	–
Mean step length (cm), mean (SD)	61.5 (9.0)	–
Mean step time (s), mean (SD)	0.6 (0.05)	–
Stance time asymmetry (s), mean (SD)	0.01 (0.01)	–
*Brain pathologies (independent variables)*
Tau pathology (SUVR, Braak stage I–IV), mean (SD)^b^	1.4 (0.4)	–
Tau SUVR, Braak stage I-II, mean (SD)^c^	1.5 (0.4)	–
Tau SUVR, Braak stage III-IV, mean (SD)^d^	1.4 (0.4)	–
Tau SUVR, Braak stage V-VI, mean (SD)^e^	1.1 (0.2)	–
Amyloid-β pathology (SUVR), mean (SD)^f^	0.7 (0.19)	–
White matter hyperintensities (mL), median (q1–q3)	6.8 (2.4–16.9)	–

BMI, body mass index; FES-I, Falls Efficacy Scale-International (16–64, higher = worse); SUVR, standardized uptake value ratio. Tau and amyloid-β were assessed with positron emission tomography, whereas white matter hyperintensities were assessed with magnetic resonance imaging. The number of steps that were registered when using an electronic walkway was in mean 39.7 (SD 7.7, min-max 30–70). ^a^Sum of right and left leg length, divided by 2. ^b^Braak stage I–IV (entorhinal cortex; inferior/middle temporal, fusiform gyrus, parahippocampal cortex, and amygdala); 37 participants (38.5%) had >1.36 SUVR, which is considered pathological. ^c^Braak stage I-II (entorhinal cortex). ^d^III-IV (inferior/middle temporal, fusiform gyrus, parahippocampal cortex, and amygdala). ^e^V-VI (widespread neocortical). ^f^Amyloid-β (composite neocortical meta region of interest: prefrontal, lateral temporal, parietal, anterior cingulate, and posterior cingulate/precuneus); 67 participants (69.8%) had >0.53 SUVR, which is considered pathological.

### Ethical statement

Ethical approval was obtained by the Regional Ethical Review Board in Lund (2016-1053) and the Swedish Ethical Review Authority (2019-02681). All participants provided written informed consent before the start of the study. Data collection was performed in accordance with the Helsinki declaration.

### Gait assessment and parameters (dependent variables)

This study focuses on gait as a single task. Collection of gait data was performed by three registered physiotherapists; all had undergone project-specific training.

All gait parameters were measured using an electronic walkway: GAITRite platinum, CIR Systems Inc. (total mat area 5.79 m long×0.89 m wide; active mat area 4.88×0.69 m; 120 Hz sampling frequency). Participants were instructed to walk at comfortable gait speed in an elliptical circuit. They walked 1.5 meters before stepping on to the electronic walkway. Approximately 1.5 meters after stepping off the walkway, participants turned around a cone and walked alongside the walkway, towards the starting point. After turning around another cone, they started their second lap. This was repeated until 6 continuous laps were completed.

A previously published principal component analysis of gait parameters in this sample generated four gait components: variability, pace/stability, rhythm, and asymmetry [3]. In this study, the highest loading gait parameter in each component (i.e., step velocity variability, step length, step time, and stance time asymmetry) were a priori chosen to be used as dependent variables in the linear regression analyses. The highest loading parameter was chosen as it is the one that most strongly influences the component.

To calculate step velocity variability, we initially calculated the variance by using all left steps and right steps separately for each person. The variance of left and right steps was then summed up and divided by two to get the mean variance, i.e., for each person. The square root of the mean variance was thereafter calculated to get step velocity variability. This approach results in a variability measure that is not affected by asymmetry between left and right steps [31]. The mean step length and step time were calculated by combining the average step parameter of each side into a total sum and then dividing it by two [32]. Stance time asymmetry was calculated as the difference (absolute value) in mean stance time between the left and right side [3]. A detailed description of the equations used to calculate the gait parameters has been provided elsewhere [3].

### Independent variables

Three brain pathology measures were used as independent variables, reflecting Aβ and tau pathology as well as WMH. All three imaging scans (i.e., Aβ-PET, tau-PET, and MRI) were performed at separate occasions and preceded gait assessments.

All participants were scanned using an MRI 3T scanner (Siemens Prisma). An automated segmentation of WMH was performed from FLAIR images using the lesion segmentation tool (LST) toolbox, implemented in SPM8 [33]. This generates an individual total lesion volume (mL), here named WMH.

PET imaging of Aβ aggregates in the brain lasted for 20 minutes and was performed 90–110 minutes following [^18^F] Flutemetamol intravenous injection. A composite neocortical meta region of interest (ROI) for Aβ pathology (prefrontal, lateral temporal, parietal, anterior cingulate, and posterior cingulate/precuneus) [34] was created using FreeSurfer software v 6.0, freely available at http://surfer.nmr.mgh.harvard.edu/. Aβ-PET load using standardized uptake value ratio (SUVR) was calculated using pons as a reference region (SUVR >0.53 = pathological).

PET imaging of tau aggregates was performed during approximately 20 min, 60 min after intravenous injection using [^18^F] RO948. A composite temporal meta ROI for tau pathology (entorhinal cortex, inferior and middle temporal cortices, fusiform gyrus, parahippocampal cortex, and amygdala) [35] was created with the assistance of FreeSurfer software v 6.0. Tau aggregate levels were calculated using the inferior cerebellar cortex as reference region [36] (Braak staging I–IV; SUVR >1.36 = pathological) [27]. To better understand tau-PET load, associations that showed statistical significance in multivariable linear regression analyses were regionally inspected, using more pathology specific regions: Braak staging I-II (entorhinal cortex); III-IV (inferior/middle temporal, fusiform gyrus, parahippocampal cortex, and amygdala); V-VI (widespread neocortical deposition) [37].

### Additional data

Age, sex, and comorbidities were used as covariates (collected at baseline of the BioFINDER-2 study, which took place shortly before the imaging scans). In models that included WMH, we also controlled for intracranial volume (ICV). Medical history (i.e., stroke, transient ischemic attack [TIA] and diabetes) was recorded through a self-administered questionnaire with questions concerning medical conditions and medications. Comorbidities were further validated by a physician examining medical records, in cases of ambiguity.

For descriptive purposes, the data collection by the physical therapists included body mass index (BMI, i.e., weight divided by height squared), leg length (also included in a sensitivity analysis), gait velocity and concerns about falling, see Table 1. Average leg length was measured as the distance from the participant’s greater trochanter to the floor while standing with their shoes on. Gait velocity was obtained by dividing the walking distance by the ambulation time (m/s). Concerns about falling was assessed using the Falls Efficacy Scale-International (FES-I); the total score ranges from 16 to 64 (higher = worse) [38].

Descriptive information included the categorization of having amnestic MCI, i.e. if they performed – 1.5 z score or worse on the delayed recall part of the Alzheimer’s Disease Assessment Scale, cognitive subscale [39]. The number and proportions of participants with pathological levels of tau and Aβ are also described in the footnotes of Table 1.

### Statistical analyses

Independent variables were tested for multicollinearity (no correlation surpassed a threshold of ±0.7). Data was checked to ensure that underlying assumptions for linear regression were met, i.e., linearity, normality and homoscedasticity [40]. One of the four dependent variables (stance time asymmetry) was square root transformed to improve normality of distribution.

Initially, univariable linear regression analyses were conducted to determine the associations between each pathology (Aβ, tau and WMH, respectively; by using continuous measures) and each of the four dependent variables: step velocity variability, step length, step time, and stance time asymmetry, see Supplementary Tables 1–4. This was followed by a series of multivariable linear regression analyses with basic covariates (i.e., adjusted for age [years], sex [0 = man, 1 = woman] and for WMH also ICV). The analyses with the pathologies and dependent variables were thereafter repeated with a time variable (number of days between imaging and gait assessment) added as a controlling variable to account for the potential effect of time differences. If the unstandardized regression coefficient (B) of any independent variable changed more than 20% when the time variable was added, a sensitivity analysis was performed. That is, the time variable was added as a controlling variable in the analysis for that specific independent variable.

Subsequent multivariable linear regression analyses included the brain pathologies that showed statistical significance in the regression analyses with basic covariates (described above). In this step, comorbidities (i.e., history of stroke/TIA [1 = yes] and diabetes [1 = yes]) were added as covariates, together with age, sex and for WMH also ICV (Table 2). For model validation, the residuals of the final multivariable models were visually inspected. That is, normally distributed residuals were validated using a histogram; linearity and homoscedasticity were validated using a scatterplot of residuals versus the different independent variables. These were also scrutinized by a senior statistician (SU, co-author), who considered all models to beacceptable.

**Table 2:** Multivariable regression analyses with step velocity variability or step length as dependent variable and tau as the independent variable (controlled for sex, age, history of stroke/transient ischemic attack and diabetes), *n* = 96

	Dependent variable					
	Step velocity variability (cm/s)			Step length (cm)		
	B (95% CI)	β	*p*	B (95% CI)	β	*p*
Tau load (SUVR) Braak I–IV	2.013 (1.01, 3.02)	0.383	**<0.001** ^a^	7.790 (3.79, 11.79)	0.336	**<0.001** ^b^

B, unstandardized regression coefficient; CI, confidence interval; β, standardized regression coefficient; SUVR, standardized uptake value ratio. Tau was assessed with positron emission tomography. The regression models included tau SUVR, according to Braak staging I–IV (entorhinal cortex, inferior/middle temporal, fusiform gyrus, parahippocampal cortex, and amygdala) along with the covariates: sex; age; history of stroke/transient ischemic attack; diabetes. Significant *p*-values (<0.05) are bolded. To account for cerebrovascular burden, additional analyses included also white matter hyperintensities (WMH, assessed with magnetic resonance imaging) and intracranial volume (ICV), i.e., ^a - b^. Adding WMH and ICV to the model: ^a^B = 1.943 (95% CI 0.92, 2.97), β= 0.369, *p* < 0.001; ^b^B = 8.112 (95% CI 4.16, 12.07), β= 0.350, *p* < 0.001.

To better understand the potential effects of tau in conventional AD-related ROIs, a number of sensitivity analyses were performed. In the complex multivariable linear regression models, regions according to different Braak stages (I-II, III-IV, and V-VI, respectively) were then used as independent variables (Table 3).

**Table 3:** Multivariable regression analyses with step velocity variability or step length as dependent variable and tau as the independent variable by using different Braak stages (controlled for sex, age, history of stroke/transient ischemic attack and diabetes), *n* = 96

	Dependent variable					
	Step velocity variability (cm/s)			Step length (cm)		
	B (95% CI)	β	*p*	B (95% CI)	β	*p*
Tau load (SUVR) Braak I-II	1.113 (0.14, 2.08)	0.229	**0.024** ^a^	6.303 (2.57, 10.04)	0.294	**0.001** ^d^
Tau load (SUVR) Braak III-IV	2.017 (1.02, 3.01)	0.386	**<0.001** ^b^	7.697 (3.71, 11.68)	0.334	**<0.001** ^e^
Tau load (SUVR) Braak V-VI	4.519 (2.44, 6.60)	0.407	**<0.001** ^c^	13.079 (4.41, 21.75)	0.267	**0.004** ^f^

B, unstandardized regression coefficient; CI, confidence interval; β, standardized regression coefficient; SUVR, standardized uptake value ratio. Tau was assessed with positron emission tomography. The regression models included Braak stage I-II (entorhinal cortex); III-IV (inferior/middle temporal, fusiform gyrus, parahippocampal cortex, and amygdala) and V-VI (widespread neocortical), respectively. Significant *p*-values (<0.05) are bolded. To account for cerebrovascular burden, additional analyses included also white matter hyperintensities (WMH, assessed with magnetic resonance imaging) and intracranial volume (ICV), i.e., ^a–f^. Adding WMH and ICV to the model: ^a^B = 1.012 (95% CI 0.003, 2.03), β= 0.209, *p* = 0.049; ^b^B = 1.948 (95% CI 0.93, 2.97), β= 0.373, *p* < 0.001; ^c^B = 4.392 (95% CI 2.28, 6.50), β= 0.395, *p* < 0.001; ^d^B = 7.072 (95% CI 3.33, 10.82), β= 0.330, *p* < 0.001; ^e^B = 7.985 (95% CI 4.05, 11.92), β= 0.347, *p* < 0.001; ^f^B = 13.354 (95% CI 4.82, 21.89), β= 0.273, *p* = 0.003.

Statistical analyses were performed using the statistical software SPSS, version 27.

## RESULTS

### Multivariable regression analyses with basic covariates

Multivariable linear regression analyses with basic covariates (Supplementary Tables 1–4) showed no significant associations between either WMH (*p*≥0.135) nor Aβ pathology (*p*≥0.090) with any of the dependent variables (i.e., the four gait parameters). Tau pathology showed a statistically significant association with step velocity variability (β= 0.378, *p* < 0.001) and step length (β= 0.337, *p* < 0.001). In the models with step time and stance time asymmetry as dependent variables (Supplementary Tables 3 and 4), none of the pathologies (tau, Aβ and WMH) showed any significant relationships with step time (*p*≥0.222) or stance time asymmetry (*p*≥0.219).

The time difference between gait assessment and imaging seemed to affect the association (adjusted for sex and age) between Aβ and stance time asymmetry in univariable analyses and in the multivariable analyses with basic covariates (i.e., the unstandardized regression coefficient changed >20% after adding the time variable as a controlling variable).

If instead using dichotomized PET-values (see footnotes in Supplementary Tables 1–4), all univariable and multivariable analyses (adjusted for age and sex) remained similar except for the association between pathological Aβ and increased step length (*p* = 0.026).

### Multivariable regression analyses that included all covariates

The multivariable linear regression models (including the independent and dependent variables that showed statistical significance in the linear regression models with basic covariates) are presented in Table 2. The first model showed that increased tau-PET load (standardized regression coefficient, β= 0.383; *p* < 0.001) was significantly and independently associated with greater step velocity variability. Using step length as the dependent variable, increased tau levels showed an effect on step length (β= 0.336; *p* < 0.001). The effect on both gait parameters remained after accounting for cerebrovascular burden. Sensitivity analyses included controlling also for leg length and gait speed, respectively. When controlling for leg length, the associations between tau and step velocity variability and step length remained statistically significant (*p* < 0.001), see Supplementary Table 5. When controlling for gait speed, the previous significant association between increased tau and increased step length became non-significant (*p* = 0.168) whereas the association with step velocity variability remained significant (*p* < 0.001), see Supplementary Table 6.

### Braak staging

Increased tau-PET load showed statistically significant associations with increased step velocity variability (*p*≤0.024) and increased step length (*p*≤0.004) also when subdividing tau load in Braak stages (I-II, III-IV, and V-VI, respectively), see Table 3. The effect of tau on step velocity variability in stage I-II was close to the predefined alpha level, when accounting for WMH (*p* = 0.049).

## DISCUSSION

This exploratory study addressed the limited knowledge regarding the independent effects of AD and cerebrovascular brain pathologies on gait parameters (from different gait components) in patients with MCI with signs of a neurodegenerative disorder. To the best of our knowledge, this MCI-study is the first to investigate the association between tau pathology and gait that also considers Aβ load and WMH. The main finding was that tau pathology is independently associated with an increased step velocity variability. Although tau pathology was initially significantly associated with increased step length, the association was non-significant when controlling for gait speed. Aβ pathology and WMH showed no significant associations with any gait parameter.

### Tau pathology

Multivariable linear regression analyses (including all covariates) showed an independent and significant association between increased tau-PET levels (Braak I–IV) and greater step velocity variability. The results remained significant in more specific AD related regions. However, the association with Braak regions I-II was close to the predefined alpha level when also accounting for WMH. This could indicate that the control of step velocity variability is more closely associated with the regions of Braak stages III-IV or V-VI.

In the present study, increased tau-PET load was associated with an increased step length in the multivariable analyses, which is counterintuitive as greater step length is considered “better”. This initial finding could be an effect of the study sample where the majority (70%) of our sample had an amnestic MCI where gait is less disturbed as compared to non-amnestic MCI [5]. Importantly, this association was no longer statistically significant when controlling for gait speed. The latter is in line with a review that highlighted the importance of controlling for gait speed when targeting parameters such as step length in older adults [41]. If using dichotomized (instead of continuous) PET values, both tau and Aβ pathology were initially associated with an increased step length. This finding became non-significant for tau-pathology when also controlling for gait speed, which corroborates our finding when using a continuous measure. Importantly, using dichotomous or continuous PET values showed similar results regarding the significant association between tau and increased step velocity variability.

The current study investigated regions of conventional spread of tau in relation to AD [13]. However, we might have found different results (both in relation to tau and Aβ pathology) if we had targeted more motor specific regions or regions previously associated with gait parameters in older adults: e.g., the pre-motor, supplementary motor and primary motor area as well as atrophy of regions related to executive function (e.g., dorsolateral prefrontal cortex) [42, 43]. Specific motor areas such as the primary motor area (for both Aβ and tau) are often affected later in the (AD) disease process [13].

### White matter hyperintensities

This study found no statistically significant associations between WMH and step velocity variability. This is in line with the study by Sakurai et al., which found no significant associations with spatiotemporal variability measures in their MCI sample (*n* = 63) [18]. Interestingly, they did find an association between WMH and stride length variability but not with step length variability; both are spatial variability measures. In the current study, we analyzed steps (i.e., instead of strides) as it has been suggested to improve the reliability of variability measures [31]. It should be noted that several studies have shown an association between WMH and gait (e.g., slower gait speed and decreased step length) in older adults, but most studies focused on gait speed [17]. Our results might have differed if we had studied WMH in more specific regions such as sensorimotor related or more cognitively specific regions, instead of using a total measure of WMH. Another potential explanation for our findings could be that the level of WMH in our sample is relatively low (median 6.8 mL). This could be compared to another study that found an association between WMH and shorter stride length; their MCI-sample had a WMH mean volume of 11.5 mL [44]. Another large study (*n* = 1,702 without dementia) reported a significant association between WMH and gait speed only among those with total WMH volumes above the 90th percentile, i.e., >10.4 mL [45]. Importantly, a recent review highlighted that two (out of our four) of the gait parameters that are explored in the present study (i.e., gait speed variability/step velocity variability and stance time asymmetry) are considered knowledge gaps both in relation to WMH and Aβ burden [17].

### Amyloid-β pathology

No significant associations were identified between Aβ PET load and the included gait parameters. In this study cohort, Aβ load might not be as closely related to clinical symptoms compared to tau load and could be a partial explanation for why tau load showed significant associations but Aβ load did not. It should be noted that some of our gait parameters are previously unexplored in relation to Aβ-PET (i.e., step velocity variability and stance time asymmetry), and the current study did not explicitly target gait speed, which is considered a global measure of gait. Two prior PET studies that showed an association between Aβ and gait speed [20, 22] had a lower proportion of Aβ-positive participants (40–48% versus 70% in our sample). Another study reported an initial significant association between global Aβ-PET and slow gait in individuals without dementia (*n* = 183), which became non-significant when adjusting for *APOE4*. The discrepancy between our study and their initial analyses might reflect that their sample was older (mean age 85.5 versus 72.4 years) and walked slower (0.88 m/s versus 1.1. m/s), but it could also reflect differences in sample sizes and the samples per se (i.e., they included both cognitively normal people and those with MCI). More importantly, we addressed step time and step velocity variability and not gait speed. Our non-significant finding of Aβ pathology in relation to step time corroborates a previous PET-study in individuals without MCI or dementia [23]. The latter study did however report an association with variability measures but only in men [23], whereas another larger study (*n* = 611) reported an association between Aβ and gait parameters only in women [19]. Importantly, prior studies present a large mixture regarding how gait was assessed, where only two [19, 23] out of the five studies used digital gait assessments. Three out of the five prior PET-studies only addressed one gait measure, i.e., gait speed [20–22]. Moreover, few studies have reported potential associations between Aβ and gait explicitly in people with MCI; this also makes comparisons difficult. Further and larger studies are needed to understand this in more depth.

None of the brain pathologies showed any significant association with step time (from the component rhythm) or stance time asymmetry (component: asymmetry), which applied for both univariable and multivariable analyses with basic covariates. This finding is congruent with a CSF study, which only reported a significant association with the rhythm component in dual task walking, i.e., not with gait assessed as a single task [24]. To the best of our knowledge, no prior study has investigated the effect of tau, Aβ or WMH on gait asymmetry parameters.

### Methodological considerations

Strengths of this study include that we target highlighted knowledge gaps within this field, i.e., the association between vascular and Alzheimer’s pathologies and gait speed variability/step velocity variability and stance time asymmetry, respectively [17]. From a clinical perspective, the included individuals are patients that frequently visit memory clinics or specialized settings relating to cognitive impairment and dementia.

The findings are of relevance for MCI-patients with signs of a neurodegenerative disorder, but the results cannot be generalized to individuals using mobility devices as they were excluded.

The cross-sectional study design is a limitation, which does not allow causal inference although imaging aspects were performed prior to gait assessment (mean 236 [SD±41.9] days). This is also the reasoning for not using the term “predictor”, which requires a longitudinal study design that preferably has more than one follow-up. It should also be noted that our sample size was somewhat limited, and future and larger studies are needed to reinforce or refute our findings.

The addition of tau-PET load might provide additional information on the effects of the included, clinically related pathology measures. In this study, tau and Aβ were assessed using PET and not CSF analysis. Although PET measures are less available and more expensive, they seem to better represent disease *stage* and more closely relate to neuropathological load and cognitive decline [46, 47]. In comparison, the less expensive and more available CSF measures can to a larger degree be regarded as disease *state* biomarkers, i.e., representing neuronal damage and neuropathology rather than intensity [46, 47]. It would be of interest to also investigate CSF measures and to target gait while dual tasking, as it stresses gait performance. Interestingly, a prior study showed that tau was not associated with mobility (i.e., Timed Up and Go) as a single task, but it was independently and significantly associated with mobility while dual tasking [48].

### Clinical implications

The independent association between increased tau depositions in AD-related brain regions and increased step velocity variability in patients with MCI is a novel finding. This indicates that specific attention should be dedicated to assessing step velocity variability when targeting gait as a single task in patients with MCI. Our findings strengthen the importance of gait variability measures, which have previously been associated with falls [49] and future cognitive decline [50]. These findings might also provide an additional step towards more profoundly understanding the association between AD pathology and gait.

### Conclusions

Our main finding is that increased tau-PET load was independently associated with increased step velocity variability in patients with MCI, who all had signs of an incipient neurodegenerative disorder. This suggests that variability measures of gait are of importance when targeting patients with MCI that have increased risk of developing dementia. The current study provides cross-sectional groundwork that could be useful for future longitudinal research that target gait parameters and the risk of developing dementia. Future studies should address additional variability measures as well as dual tasking in larger cohorts to further disentangle the relationship between AD pathology and gait disturbances suggested by this explorative study.

## Supplementary Material

Supplementary Material

## Data Availability

The data supporting the findings of this study are available on request from the corresponding author. The data are not publicly available due to privacy or ethical restrictions.
