# Supplementary material for: Effects of Brain Pathologies on Spatiotemporal Gait Parameters in Patients with Mild Cognitive Impairment
Source: J Alzheimers Dis. 2023 Oct 24;96(1):161–71. doi: 10.3233/JAD-221303 (PMC10657715; doi:10.3233/JAD-221303)
Supplement: Supplementary Material [file jad-96-jad221303-s001.pdf]

Supplementary Material

Effects of Brain Pathologies on Spatiotemporal Gait Parameters in Patients with Mild Cognitive Impairment

METHODS

Imaging

All three imaging scans were performed within 6 months for all but one patient, who had a little bit less than 7 months (209 days) between MRI and tau-PET, and 8 months (246 days) between tau-PET and Aβ-PET. However, the vast majority of the sample had their three scans within a rather short time period. The mean number of days between the scans were as follows:

- MRI and tau-PET: mean 8 days
- MRI and Aβ-PET: mean 20 days
- Tau-PET and Aβ-PET: mean 12 days

Scatter plots

The unadjusted associations between the tau PET (SUVR) variable (i.e., Braak I-IV) and the gait parameters are presented below.

Supplementary Figure 1. Unadjusted association between tau PET (SUVR, Braak I-IV) and step velocity variability (cm/s).

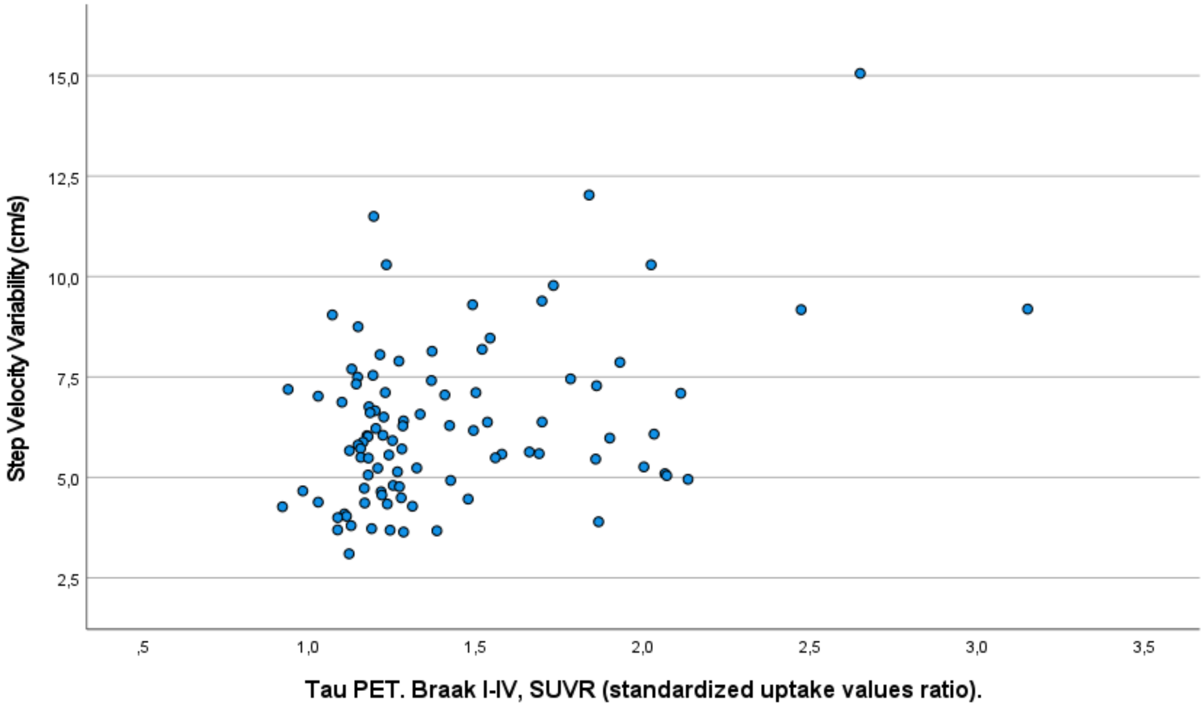

Supplementary Figure 2. Unadjusted association between Tau PET (SUVR, Braak I-IV) and step length (cm).

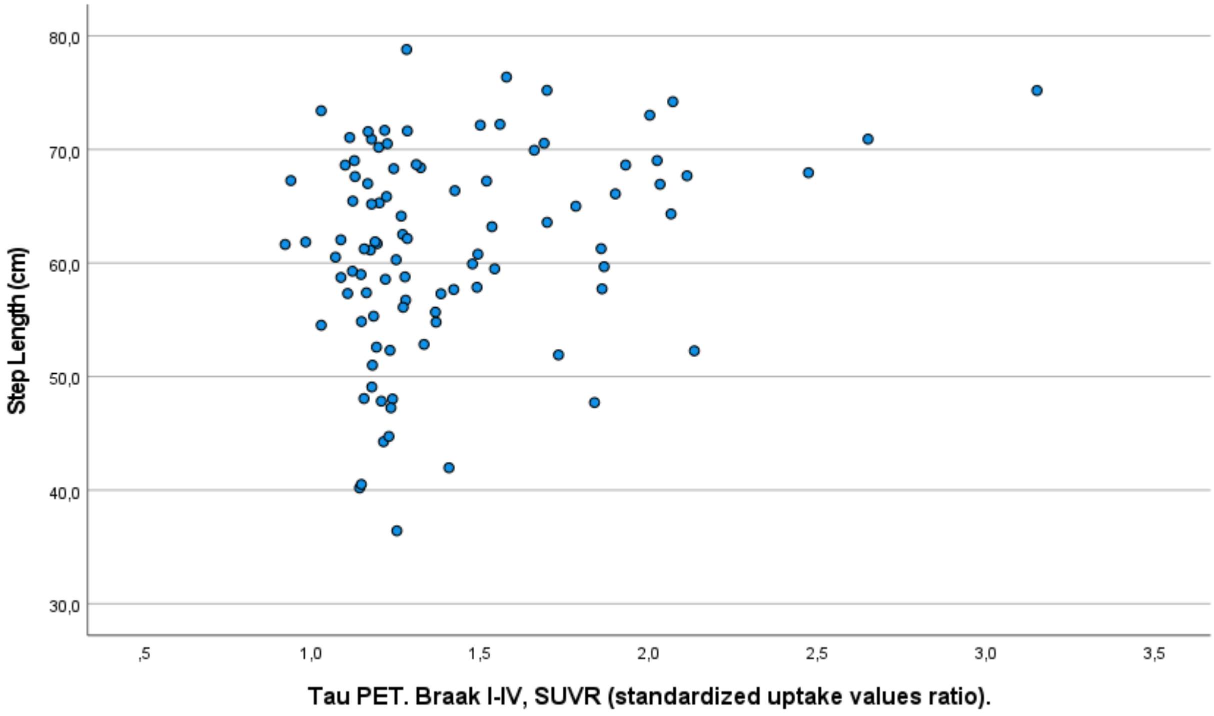

Supplementary Figure 3. Unadjusted association between Tau PET (SUVR, Braak I-IV) and step times (s).

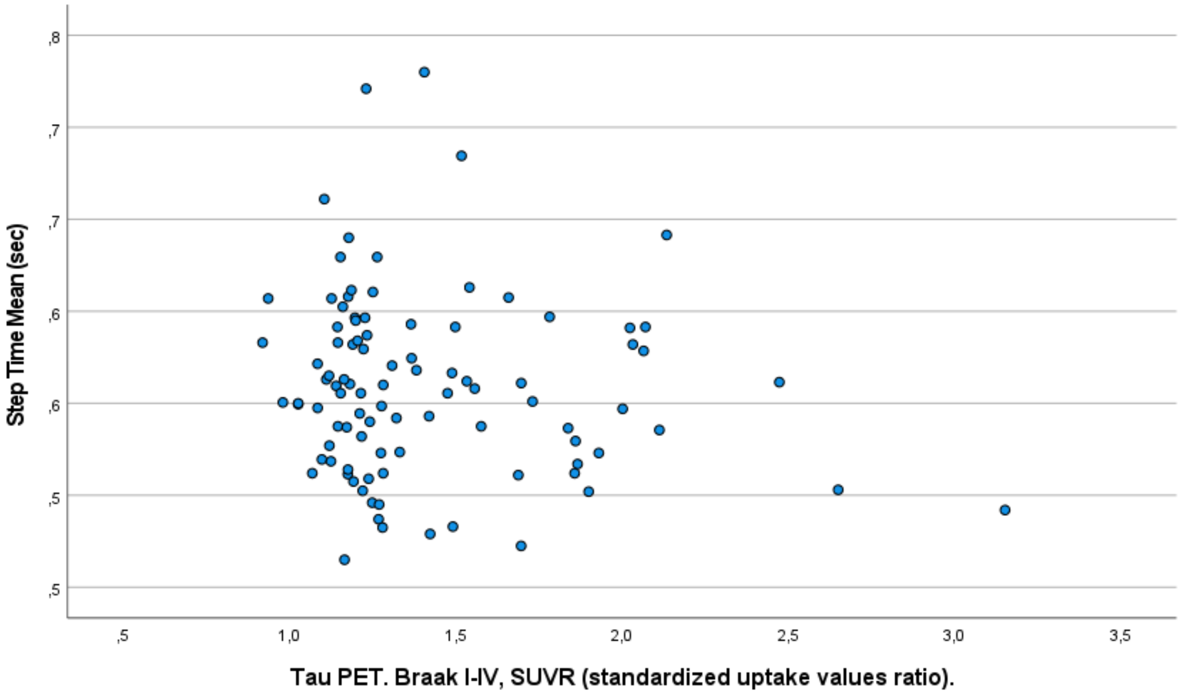

Supplementary Figure 4. Unadjusted association between Tau PET (SUVR, Braak I-IV) and stance time asymmetry (s, square root transformed).

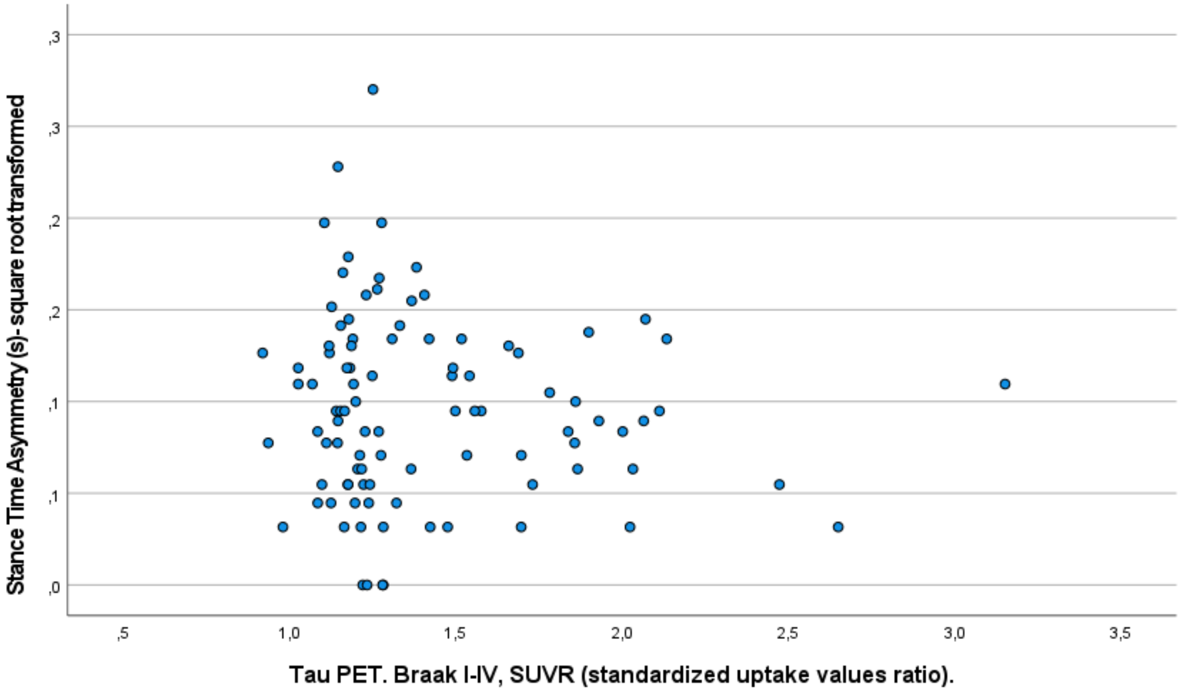

Supplementary Table S1. Univariable and multivariable linear regression analyses with step velocity VARIABILITY (cm/s) as the dependent variable; separate models for each pathology variable (tau, amyloid-β, and white matter hyperintensities, respectively), n = 96

Univariable (unadjusted)			
Independent variable	B (95% CI)	β	p
Tau load (SUVR), Braak stage I-IV[a]	2.085 (1.09, 3.08)	0.396	**<0.001**
Amyloid-β (SUVR)[b]	-0.219 (-2.47, 2.04)	-0.020	0.847
White matter hyperintensities (mL)	-0.014 (-0.04, 0.02)	-0.116	0.261
Multivariable (adjusted for age and sex + intracranial volume for white matter hyperintensities)			
	B (95% CI)	β	p
Tau load (SUVR), Braak stage I-IV[c]	1.988 (0.98, 3.00)	0.378	**<0.001**
Amyloid-β (SUVR)[d]	-0.383 (-2.67, 1.91)	-0.035	0.740
White matter hyperintensities (mL)	-0.019 (-0.05, 0.01)	-0.160	0.135

B, unstandardized regression coefficient; CI, confidence interval; β, standardized regression coefficient; SUVR, standardized uptake value ratio.

Tau and amyloid-β were assessed using positron emission tomography. White matter hyperintensities were assessed using magnetic resonance imaging. Significant p-values (<0.05) are bolded.

When instead using dichotomized tau PET (>1.36 SUVR = pathological) and amyloid-β PET (>0.53 SUVR = pathological); 1 = pathological:

[a] B = 1.269 (95% CI 0.45, 2.09), β = 0.304, p = 0.003

[b] B = -0.158 (95% CI -1.07, 0.75), β = -0.036, p = 0.729

[c] B = 1.240 (95% CI 0.42, 2.06), β = 0.297, p = 0.003

[d] B = -0.298 (95% CI -1.23, 0.63), β = -0.067, p = 0.525

Supplementary Table 2. Univariable and multivariable linear regression analyses with step LENGTH (cm) as the dependent variable; separate models for each pathology variable (tau, amyloid-β and white matter hyperintensities, respectively), n = 96

Univariable (unadjusted)			
Independent variable	B (95% CI)	β	p
Tau load (SUVR), Braak stage I-IV[a]	6.496 (1.93, 11.06)	0.280	**0.006**
Amyloid-β (SUVR)[b]	3.020 (-6.88, 12.92)	0.062	0.546*
White matter hyperintensities (mL)	-0.097 (-0.21, 0.01)	-0.182	0.076
Multivariable (adjusted for age and sex + intracranial volume for white matter hyperintensities)			
	B (95% CI)	β	p
Tau load (SUVR), Braak stage I-IV[c]	7.827 (3.88, 11.78)	0.337	**<0.001**
Amyloid-β (SUVR)[d]	7.646 (-1.21, 16.50)	0.158	0.090
White matter hyperintensities (mL)	-0.059 (-0.16, 0.04)	-0.111	0.239

B, unstandardized regression coefficient; CI, confidence interval; β, standardized regression coefficient; SUVR, standardized uptake value ratio.

Tau and amyloid-β were assessed using positron emission tomography. White matter hyperintensities were assessed using magnetic resonance imaging. Significant p-values (<0.05) are bolded.

* The unstandardized regression coefficient changed >20% when a time difference variable was added (p-value was then 0.447).

When instead using dichotomized tau PET (>1.36 SUVR = pathological) and amyloid-β PET (>0.53 SUVR = pathological); 1 = pathological:

[a] B = 3.844 (95% CI 0.16, 7.53), β = 0.209, p = 0.041

[b] B = 1.505 (95% CI -2.47, 5.49), β = 0.077, p = 0.455

[c] B = 4.488 (95% CI 1.26, 7.71), β = 0.244, p = 0.007

[d] B = 4.043 (95% CI 0.48, 7.61), β = 0.207, p = 0.026

Supplementary Table 3. Univariable and multivariable linear regression analyses with step TIME (s) as the dependent variable; separate models for each pathology variable (tau, amyloid-β and white matter hyperintensities, respectively), n = 96

Univariable (unadjusted)			
Independent variable	B (95% CI)	β	p
Tau load (SUVR), Braak stage I-IV[a]	-0.017 (-0.05, 0.01)	-0.130	0.208
Amyloid-β (SUVR)[b]	0.032 (-0.03, 0.09)	0.119	0.248
White matter hyperintensities (mL)	-0.000 (-0.01, 0.01)	-0.023	0.827
**Multivariable (adjusted for age and sex + intracranial volume for white matter hyperintensities)**			
	B (95% CI)	β	p
Tau load (SUVR), Braak stage I-IV[c]	-0.015 (-0.04, 0.01)	-0.119	0.222
Amyloid-β (SUVR)[d]	0.015 (-0.04, 0.07)	0.054	0.583
White matter hyperintensities (mL)	0.000 (-0.01, 0.000)	-0.046	0.654

B, unstandardized regression coefficient; CI, confidence interval; β, standardized regression coefficient; SUVR, standardized uptake value ratio.

Tau and amyloid-β were assessed using positron emission tomography. White matter hyperintensities were assessed using magnetic resonance imaging. Significant p-values (<0.05) are bolded.

When instead using dichotomized tau PET (>1.36 SUVR = pathological) and amyloid-β PET (>0.53 SUVR = pathological); 1= pathological:

[a] B = -0.001 (95% CI -0.03, 0.02), β = -0.006, p = 0.957

[b] B = 0.016 (95% CI -0.01, 0.04), β = 0.147, p = 0.154

[c] B = -0.002 (95% CI -0.03, 0.02), β = -0.023, p = 0.814

[d] B = 0.009 (95% CI -0.02, 0.04), β = 0.088, p = 0.379

Supplementary Table 4. Univariable and multivariable linear regression analyses with stance time ASYMMETRY* (s) as the dependent variable; separate models for each pathology variable (tau, amyloid-β and white matter hyperintensities, respectively), n = 96

Univariable (unadjusted)			
Independent variable	B (95% CI)	β	p
Tau load (SUVR), Braak stage I-IV[a]	-0.012 (-0.04, 0.02)	-0.091	0.377
Amyloid-β (SUVR)[b]	0.011 (-0.05, 0.07)	0.039	0.704**
White matter hyperintensities (mL)	0.000 (-0.01, 0.000)	-0.065	0.527
Multivariable (adjusted for age and sex + intracranial volume for white matter hyperintensities)			
	B (95% CI)	β	p
Tau load (SUVR), Braak stage I-IV[c]	-0.013 (-0.04, 0.02)	-0.101	0.308
Amyloid-β (SUVR)[d]	-0.009 (-0.07, 0.05)	-0.033	0.743***
White matter hyperintensities (mL)	0.000 (-0.01, 0.000)	-0.126	0.219

B, unstandardized regression coefficient; CI, confidence interval; β, standardized regression coefficient; SUVR, standardized uptake value ratio.

Tau and amyloid-β were assessed using positron emission tomography. White matter hyperintensities were assessed using magnetic resonance imaging. Significant p-values (<0.05) are bolded.

* Square root transformed. ** The unstandardized regression coefficient changed >20% when a time difference variable was added (p-value was then 0.833). *** The unstandardized regression coefficient changed >20% when a time difference variable was added (p-value was then 0.681). When instead using dichotomized tau PET (>1.36 SUVR = pathological) and amyloid-β PET (>0.53 SUVR = pathological); 1 = pathological:

[a] B = -0.003 (95% CI -0.03, 0.02), β = -0.028, p = 0.789

[b] B = 0.003 (95% CI -0.02, 0.03), β = 0.028, p = 0.790

[c] B = -0.005 (95% CI -0.03, 0.02), β = -0.050, p = 0.614

[d] B = 0.006 (95% CI -0.03, 0.02), β = -0.054, p = 0.596

Supplementary Table 5. Leg length was added as a covariate. Multivariable regression analyses with step velocity variability or step length as dependent variable and tau as the independent variable (controlled for leg length, sex, age, history of stroke/transient ischemic attack and diabetes), n = 96

	Dependent variable					
	Step velocity variability (cm/s)			Step length (cm)		
	B (95% CI)	β	p	B (95% CI)	β	p
Tau load (SUVR)						
Braak I-IV	1.963 (0.96, 2.96)	0.373	**<0.001**[a]	7.439 (3.54, 11.34)	0.321	**<0.001**[b]

B, unstandardized regression coefficient; CI, confidence interval; β, standardized regression coefficient; SUVR, standardized uptake value ratio. Tau was assessed with positron emission tomography. The regression models included tau SUVR, according to Braak staging I-IV (entorhinal cortex, inferior/middle temporal, fusiform gyrus, parahippocampal cortex, and amygdala) along with the covariates: leg length; sex; age; history of stroke/transient ischemic attack; diabetes. Significant p-values (<0.05) are bolded. To account for cerebrovascular burden, additional analyses included also white matter hyperintensities (WMH, assessed with magnetic resonance imaging) and intracranial volume (ICV), i.e., [a-b].

Adding WMH and ICV to the model: [a] B = 1.885 (95% CI 0.86, 2.91), β = 0.358, p <0.001;

[b] B = 7.729 (95% CI 3.88, 11.58), β = 0.333, p <0.001.

Supplementary Table 6. Step velocity was added as a covariate. Multivariable regression analyses with step velocity variability or step length as dependent variable and tau as the independent variable (controlled for step velocity, sex, age, history of stroke/transient ischemic attack and diabetes), n = 96

	Dependent variable					
	Step velocity variability (cm/s)			Step length (cm)		
	B (95% CI)	β	p	B (95% CI)	β	p
Tau load (SUVR)						
Braak I-IV	2.164 (1.09, 3.24)	0.411	**<0.001**[a]	1.466 (-0.62, 3.56)	0.063	0.168[b]

B, unstandardized regression coefficient; CI, confidence interval; β, standardized regression coefficient; SUVR, standardized uptake value ratio. Tau was assessed with positron emission tomography. The regression models included tau SUVR, according to Braak staging I-IV (entorhinal cortex, inferior/middle temporal, fusiform gyrus, parahippocampal cortex, and amygdala) along with the covariates: step velocity; sex; age; history of stroke/transient ischemic attack; diabetes. Significant p-values (<0.05) are bolded. To account for cerebrovascular burden, additional analyses included also white matter hyperintensities (WMH, assessed with magnetic resonance imaging) and intracranial volume (ICV), i.e., [a-b]. Adding WMH and ICV to the model: [a] B = 2.106 (95% CI 0.99, 3.22), β = 0.400, p <0.001; [b] B = 1.524 (95% CI -0.61, 3.66), β = 0.066, p = 0.159.
